# 995. A Murine Model of *Klebsiella pneumoniae* Gastrointestinal Colonization with Parenteral Vancomycin Administration

**DOI:** 10.1093/ofid/ofab466.1189

**Published:** 2021-12-04

**Authors:** Bettina Cheung, Marine Lebrun-Corbin, Alan R Hauser

**Affiliations:** Northwestern University, Chicago, Illinois

## Abstract

**Background:**

As a leading cause of nosocomial infections, *Klebsiella pneumoniae* poses a significant threat due to its propensity to acquire resistance to many classes of antibiotics, including carbapenems. Gastrointestinal (GI) colonization by *K. pneumoniae* is a risk factor for subsequent infection as well as transmission to other patients. To study this crucial step in pathogenesis, we developed a mouse model of *K. pneumoniae* GI colonization using a clinically relevant parenteral antibiotic regimen.

**Methods:**

To improve the clinical relevance of our model, we elected to use intraperitoneal injections of vancomycin, one of the most highly utilized antibiotics in the United States.

**Results:**

To optimize dosage in C57bl/6 mice, we injected 20mg/kg, 350mg/kg, or vehicle (PBS) for three days prior to gastric gavage with 10^8^ colony forming units (CFU) of a low-resistance strain of *K. pneumoniae*. The mice who received 350mg/kg (a mouse equivalent of a human dose of 1g/day calculated through the FDA guidelines for estimating safe dosing) shed about 10^7^ CFU/g of feces at Day 7 while those receiving the lower dose or vehicle shed 10^4^ CFU/g. Next, we compared 3- or 5-day pre-treatments with vancomycin prior to inoculation with an ST258 (epidemic carbapenem-resistant) strain. At Day 7 post-inoculation, mice who received 5 days shed 10^10^ CFU/g feces while those who received vancomycin for 3 days or vehicle for 5 days (PBS) shed 10^6^ or 10^4^ CFU/g feces respectively. Thus, we chose 5 days of 350mg/kg vancomycin injection as our regimen for inducing robust GI colonization in mice. Finally, we tested the durability of colonization by following fecal shedding in mice up to Day 60 post-inoculation with a second ST258 strain. Shedding during the first 7 days occurs at about 10^10^ CFU/g feces, and from day 14 to day 60 fecal loads are stable around 10^7^ CFU/g feces. Results are comparable between male and female mice.

**Conclusion:**

In conclusion, we have developed a mouse model of robust, prolonged GI colonization with multiple strains of *K. pneumoniae* using controlled dosing of a clinically relevant antibiotic. This model may be used to study a key step in *K. pneumoniae* pathogenesis and infection prevention in the future.

**Disclosures:**

**All Authors**: No reported disclosures

